# Prognosis of Adults With Isolated Left Ventricular Non-Compaction: Results of a Prospective Multicentric Study

**DOI:** 10.3389/fcvm.2022.856160

**Published:** 2022-05-02

**Authors:** Hilla Gerard, Nicolas Iline, Hélène Martel, Karine Nguyen, Pascale Richard, Erwan Donal, Jean-Christophe Eicher, Olivier Huttin, Christine Selton-Suty, Pascale Raud-Raynier, Guillaume Jondeau, Nicolas Mansencal, Caroline Sawka, Flavie Ader, Jean-François Pruny, Anne-Claire Casalta, Nicolas Michel, Valeria Donghi, Laurence Faivre, Roch Giorgi, Philippe Charron, Gilbert Habib

**Affiliations:** ^1^Cardiology Department, Assistance Publique Hopitaux de Marseille (APHM), La Timone Hospital, Marseille, France; ^2^Assistance Publique Hopitaux de Marseille (APHM), Hop Timone, BioSTIC, Biostatistique et Technologies de l'Information et de la Communication, Marseille, France; ^3^Département de génétique médicale, Assistance Publique Hopitaux de Marseille (APHM), Hôpital d'enfants de la Timone, Marseille, France; ^4^Aix Marseille University, INSERM, Marseille Medical Genetics, Faculté de Médecine, Marseille, France; ^5^Assistance Publique Hopitaux de Paris (APHP), Functional Unit of Cardiogénétique et Myogénétique, Service de Biochimie Métabolique, Hôpitaux Universitaires de la Pitié-Salpêtrière-Charles Foix, Paris, France; ^6^Sorbonne Universités, INSERM, UMR_S 1166 and ICAN Institute for Cardiometabolism and Nutrition, Paris, France; ^7^Service de Cardiologie, Centre Hospitalier Régional Universitaire Pontchaillou, Rennes, France; ^8^Service de Cardiologie, CHU Dijon Bourgogne - Hôpital François Mitterrand, Dijon, France; ^9^Service de Cardiologie, CHU de Nancy, Hôpitaux de Brabois, Vandoeuvre-lès-Nancy, France; ^10^Service de Cardiologie, CHU de Poitiers, Poitiers, France; ^11^Assistance Publique Hopitaux de Paris (APHP), Service Cardiologie, CHU Paris Nord- Val de Seine - Hôpital Xavier Bichat-Claude-Bernard, Paris, France; ^12^Assistance Publique Hopitaux de Paris (APHP), Service de Cardiologie, CHU Ambroise Paré, Boulogne Billancourt, France; ^13^Centre de génétique et FHU TRANSLAD, Hôpital d'Enfants et Université de Bourgogne, Dijon, France; ^14^Assistance Publique Hopitaux de Paris (APHP), Centre de Référence pour les Maladies Cardiaques Héréditaires, Hôpital Pitié- Salpêtrière, Paris, France; ^15^Aix Marseille Univ, Assistance Publique Hopitaux de Marseille (APHM), INSERM, IRD, SESSTIM, Sciences Economiques & Sociales de la Santé & Traitement de l'Information Médicale, ISSPAM, Hop Timone, BioSTIC, Biostatistique et Technologies de l'Information et de la Communication, Marseille, France; ^16^Aix Marseille Univ, IRD, Assistance Publique Hopitaux de Marseille (APHM), MEPHI, IHU-Méditerranée Infection, Marseille, France

**Keywords:** left ventricular non-compaction, prognosis, dilated cardiomyopathy registry, registry, heart failure

## Abstract

**Background:**

Whether left ventricular non-compaction (LVNC) bears a different prognosis than dilated cardiomyopathy (DCM) is still a matter of debate.

**Methods:**

From a multicenter French prospective registry, we compared the outcomes of 98 patients with LVNC and 65 with DCM. The primary endpoint combined cardiovascular death, heart transplantation, and hospitalization for cardiovascular events. The two groups presented similar outcomes but different left ventricular ejection fractions (LVEF) (43.3% in LVNC vs. 35.95% in DCM, *p* = 0.001). For this reason, a subgroup analysis was performed comparing only patients with LVEF **≤** 45%, including 56 with LVNC and 49 with DCM.

**Results:**

Among patients with LVEF**≤** 45%, at 5-year follow-up, the primary endpoint occurred in 33 (58.9%) among 56 patients with LVNC and 18 (36.7%) among 49 patients with DCM (*p* = 0.02). Hospitalization for heart failure (18 [32.14%] vs. 5 [10.20%], *p* = 0.035) and heart transplantation were more frequent in the LVNC than in the DCM group. The incidences of rhythmic complications (24 [42.85%] vs. 12 [24.48%], *p* = 0.17), embolic events, and cardiovascular death were similar between LVNC and DCM cases. Among the 42 patients with LVNC and LVEF > 45%, the primary endpoints occurred in only 4 (9.52%) patients, including 2 hospitalizations for heart failure and 3 rhythmic complications, but no embolic events.

**Conclusion:**

In this prospective cohort, patients with LVNC who have left ventricular dysfunction present a poorer prognosis than DCM patients. Heart failure events were especially more frequent, but embolic events were not. Patients with LVNC and preserved ejection fraction present very few events in 5 years.

## Introduction

Left ventricular non-compaction (LVNC) is a rare cardiomyopathy characterized by the association of prominent trabeculations and deep recesses in the left ventricle (1–7). This form of cardiomyopathy has long been difficult to classify (1), and whether LVNC is a distinct cardiomyopathy or a morphologic trait has even been questioned (8–10). DCM has an estimated prevalence of one case in 2,500 individuals, is a major cause of heart failure, and encompasses a broad range of underlying causes, with a growing proportion of familial/genetic causes (11). Conversely, the epidemiology and genetic characteristics of LVNC are less well defined, mainly because different diagnostic criteria have been used (1–4). However, recent genetic studies clearly identified LVNC as a specific genetic disease, with a genetic profile different from that of DCM (12).

The prognosis of LVNC also differed between previous studies, ranging from young populations of patients with the malignant course (7, 8) to the more recently reported asymptomatic phenotypes (9, 10). Finally, whether LVNC bears a different prognosis than dilated cardiomyopathy (DCM) is still a matter of debate.

To answer this question, we initiated a prospective multicenter French registry for patients newly diagnosed with LVNC between 2012 and 2014. The genetic defects carried by those patients were recently published (12), as well as their phenotype/genotype relationship (13). This study was conducted as part of the French Programme Hospitalier de Recherche Clinique (PHRC Ref: 2011-A-00987-34) aiming at comparing the prognosis of a new prospective cohort of LVNC with matched patients with dilated cardiomyopathy.

We report here the results of this prospective study, focusing on the respective outcomes of the populations of LVNC and DCM.

## Materials and Methods

### Study Population

From 2012 to 2014, patients newly diagnosed with LVNC or DCM in 9 French centers for inherited cardiac diseases were prospectively included. Inclusion criteria: all patients above the age of 18 who were newly diagnosed (<6 months) with isolated LVNC or idiopathic DCM by TTE ± MRI more than 1 month after an episode of acute heart failure. We applied the Jenni criteria for LVNC selection (2): the presence of left ventricular (LV) trabeculations and deep recesses in communication with the LV cavity, as well as a ratio of NC/C > 2 in systole. The left ventricular ejection fraction was systematically measured using the Simpson biplane rule. Assessment of left ventricular longitudinal function by echocardiographic global longitudinal strain (GLS) was also reported when available. Cardiac MRI was also performed in 70 (71%) of the 98 patients with LVNC in our series, with an NC/C ratio of >2.3 in diastole as the recommended threshold for the diagnosis of LVNC using this technique (14). However, as previously reported (12), for the purpose of this study, only the echocardiographic criteria were used as inclusion criteria, with MRI confirmation in most cases. Finally, only patients with a validated diagnosis of LVNC confirmed were included. DCM was defined according to the ESC Working Group on Myocardial and Pericardial Diseases report (1) that accounts for LV or biventricular systolic dysfunction and dilatation that are not explained by abnormal loading conditions or coronary artery disease. All TTE results were independently validated by a core lab centralized review before inclusion. Underage patients, prevalent cases, valvular, ischemic, or congenital heart disease were excluded. This study complies with the Declaration of Helsinki and was approved by our institutional review board. Written informed consent was obtained from all patients. Participating centers managed the approvals of their ethics committees or Institutional Review Boards, according to local regulations.

Patients with LVNC also underwent a genetic analysis, as an ancillary study of the main registry (12).

### Data Collection

All patients underwent a comprehensive baseline clinical and echocardiographic study with core lab analysis and genetic analysis. In addition to standard measurements, echocardiographic studies focused on trabecular quantification, extent, and localization. All TTE examinations were anonymized and transmitted to the main investigation center (Cardiology Department of the Timone Hospital, Marseille). A specific, unique code was used to identify each of the centers and patients. Demographics, comorbidities, and laboratory data were collected at baseline and at 1 and 2 years of follow-up. All patients were subjected to a physical examination and electrocardiography at the time of enrollment, which was repeated after 1 and 2 years. The scheduled end of the study visit was the 2-year follow-up visit for surviving patients. For deceased or transplanted ones, the last visit before the event was considered the end of the study visit. Data about the type of event and date of occurrence were obtained from patients' or clinicians' reports. A 5-year follow-up was also obtained through phone calls or contact with the patient or his practitioner.

### Primary and Secondary Endpoints

The primary endpoint was the occurrence of cardiac death, heart transplantation, or hospitalization for cardiovascular complications (hemodynamic, rhythmic, or embolic) during follow-up. An initial follow-up was scheduled at 2 years, and then completed by a final evaluation at 5 years. Hemodynamic events were defined as the occurrence of pulmonary edema or hospitalization for acute heart failure. Embolic events include the occurrence of a stroke or of clinically detectable embolism in any territory requiring hospitalization. Rhythmic events included sustained ventricular tachycardia, syncope, or ICD-appropriate shock, as well as permanent or paroxysmal atrial fibrillation. Implantation of a pacemaker or defibrillator was also collected.

Secondary endpoints were the occurrence of any of the events that were comprised in the primary outcome.

### Analysis of Patients With Reduced LVEF

**Figure 1:**
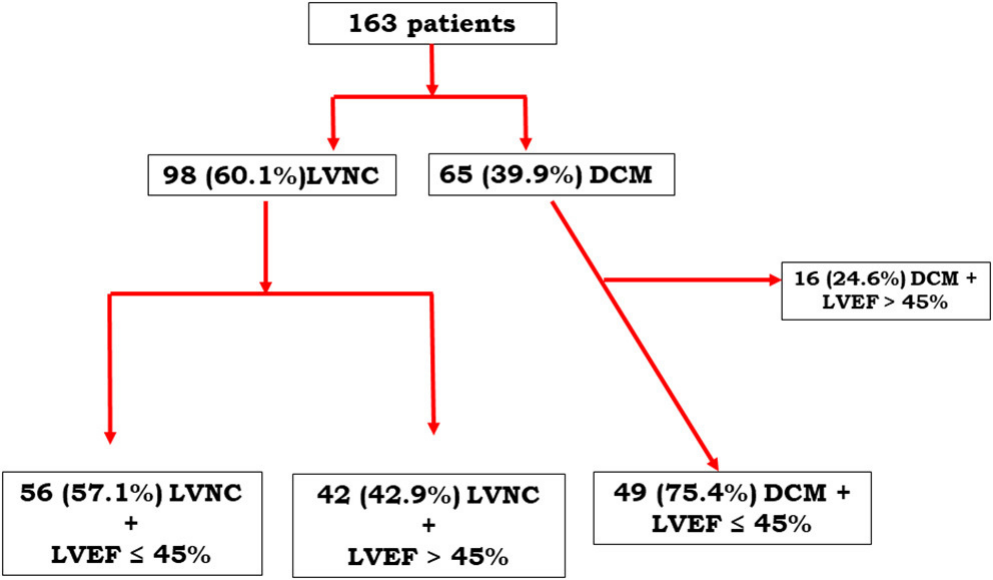
Flow chart.

To reduce the influence of LVEF on prognosis, a specific substudy was performed, including only patients with LVEF <45%, following the recent definition of DCM given by a position statement of the ESC working group on myocardial and pericardial diseases (15) and the expert consensus document from the European Association of Cardiovascular Imaging (11). Figure 1 summarizes the flow chart of the registry.

### Statistical Analysis

Continuous variables were expressed as mean and standard deviation and were compared using the Mann–Whitney nonparametric U test or Student's *t-*test, depending on the application conditions. Categorical variables were described by their number and percentage and were compared using the chi-squared test or the Fisher test, depending on the application conditions. Event-free survival distributions were estimated using the Kaplan–Meier method. The log-rank test was used to compare survival distributions for categorical variables, such as the cardiomyopathy variable. The effect of continuous variables on the risk of an event occurring was estimated and tested using a Cox model. Multivariate analyses were performed using the Cox model. Inclusion of variables in the fitted model was carried out according to the following criteria: *p* < 0.20 in univariate analysis as the threshold for entering variables, number of missing values <10% for these variables, effective presence in both categories of a bivariate variable >10%, and according to their clinical relevance. The final models selected were those with the lowest Akaike information criteria (AIC). Tests were performed in bilateral situations and were considered statistically significant for *p* < 0.05. Statistical analysis was performed using R software (version 3.6.2) and RStudio (version 1.2.5).

## Results

### Clinical and Echocardiographic Characteristics at Baseline

Between January 2012 and February 2014, a total of 98 LVNC and 65 DCM cases were prospectively included. The clinical and echocardiographic characteristics of the study population are summarized in Table 1. Age at diagnosis was similar between groups. No significant difference was found over symptoms except for the occurrence of syncope, which was more frequently reported in LVNC cases (*p* = 0.05). DCM patients presented with more severe LV dysfunction, including higher end-diastolic and end-systolic volumes and lower LVEF (43.3% [15–71] vs. 35.95% [15–55.7], *p* = 0.001). Concerning the LVNC group, mutations were identified in 47 (48%) patients, including sarcomeric mutations in 23 (48.9%) (10 TTN, 3 MYH6, 3 MYH7, 2 MYBPC3, 3 ACTC1, and 2 other sarcomeric genes) and ion-channel mutations in 15 (32% of mutated patients) (8 patients with mutations in HCN4 and 7 in RYR2).

**Table 1:** LVNC and DCM clinical and echocardiographic characteristics at inclusion.

	**LVNC *N* = 98**	**DCM** ***N* = 65**	***p*-value**
Clinical features			
Age (median and IQR), years	46.6 (18–81)	49.4 (21–84)	0.31
Male sex (n, %)	58.1 (59.2)	40.0 (61.6)	0.19
Heart Rate (bpm)	68.8 [36–127]	70.4[48–108]	0.53
NYHA (*n*, %) NYHA I NYHA II NYHA III NYHA IV	51 (52.0) 39 (39.8) 6 (6.1) 2 (2.0)	22 (32.8) 34 (52.3) 8 (12.3) 1 (1.54)	0.09
Palpitations (*n*, %)	23 (23.5)	15 (23.0)	0.95
Chest pain (*n*, %)	12 (12.2)	6 (9.2)	0.55
Syncope (*n*, %)	10 (10.2)	1 (1.5)	0.05
Sudden cardiac death among family members (*n*, %)	10 (10.2)	4 (6.1)	0.37
TTE
LVEDD (mm)	58.6 [33–82]	64.8 [52–80]	<0.001
LVESD (mm)	45.8 [17.9–75]	52.8 [39–75]	<0.001
LA diameter (mm)	41.2 [23–60]	40.8 [29–63]	0.778
LA volume (ml)	71.8 [22.7–170]	69.7 [22–163]	0.937
LVEF (%)	43.3 [15–71]	35.9 [15–55.7]	0.001
LVEDV (ml)	158.9 [34–360]	195.3 [90–395]	0.002
LVESV (ml)	95.4 [23–279]	130.9 [53–298]	<0.001
High Filling Pressures (n, %)	21 (26.6%)	15 (24.5%)	0.789
Cardiac Index l/mn/m^2^	2.55 [1.15–6.38]	2.4 [1.37–4.6]	0.399

*LVEF, left ventricular ejection fraction; LVEDD, left ventricular end-diastolic diameter; LVESD, left ventricular end-systolic diameter; LA, left atrium; LVEDV, left ventricular end-diastolic volume; LVESV, left ventricular end-systolic volume*.

### Outcome of the 98 LVNC and 65 DCM Cases

**Figure 2:**
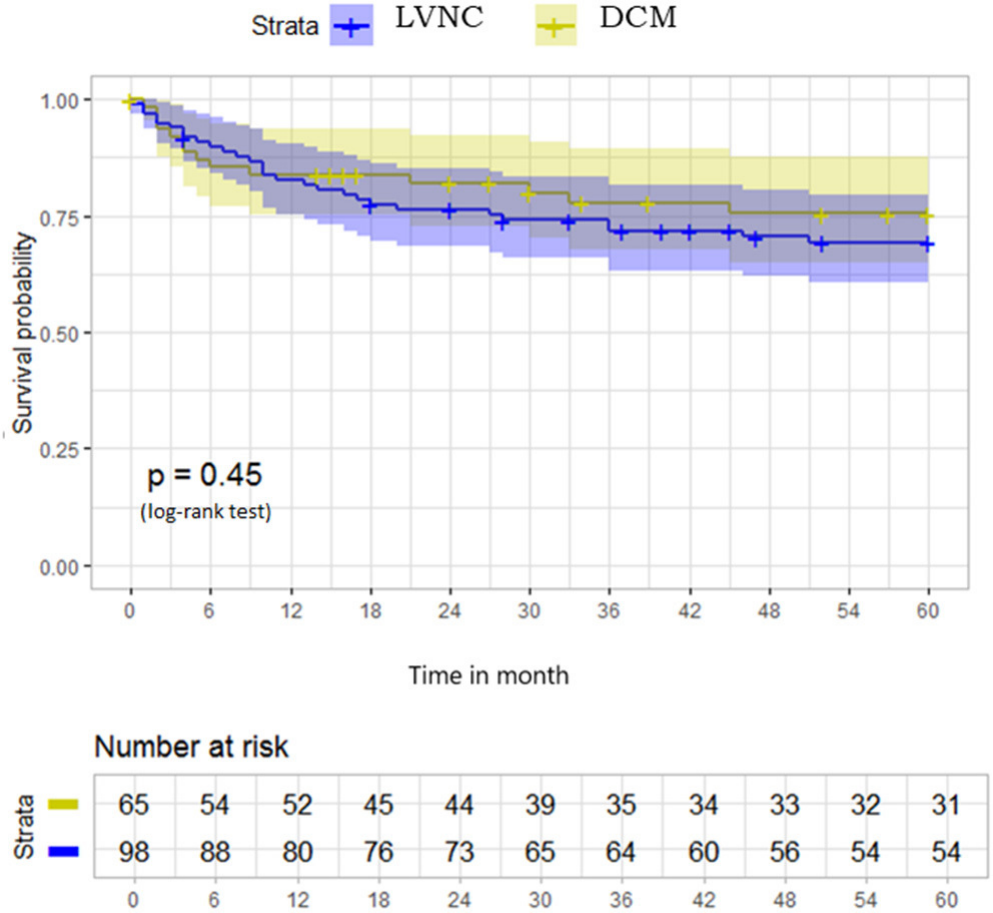
Survival free from primary outcome at 5-year follow-up in the whole population of 98 LVNC and 65 DCM cases. LVNC, left ventricular non-compaction; DCM, dilated cardiomyopathy.

The mean follow-up was 18.2 months for patients with LVNC and 17.8 months for patients with DCM, with 8 and 10 patients lost to follow-up, respectively. At 2-year follow-up, the primary endpoint was reached in 22 (22.4%) LVNC and 12 (17.9%) DCM cases, *p* = 0. 57. The mean 5-year follow-up was 73.69 months. The Kaplan–Meier curve in Figure 2 illustrates that the two groups' event-free survival was similar at a 5-year follow-up.

### Outcome of Patients With LVEF <45%

To compare patients with similar LVEF, we compared the prognosis of patients with LVEF <45% in both groups, including 56 patients with LVNC and 49 patients with DCM. No significant difference was observed in demographics or TTE parameters between the two groups (Table 2).

**Table 2:** Clinical and echocardiographic features of LVNC with LVEF <45% and DCM with LVEF <45%.

**Clinical features**	**LVNC with EF <45% *N* = 56**	**DCM with EF <45%** ***N* = 49**	***p*-value**
Age (median and IQR), years	51.5 [19–81]	51.6 [23–84]	0.94
Male sex (n, %)	34 (60.7)	36 (70.5)	0.283
Heart Rate (bpm)	69.8 [44–119]	70.1 [48–108]	0.89
NYHA (n, %) NYHA I NYHA II NYHA III NYHA IV	21 (37.5) 28 (50.0) 5 (8.9) 2 (3.6)	14 (28.5) 26 (53.1) 8 (16.3) 1 (2.0)	0.58
Palpitations (*n*, %)	11 (19.6)	11 (22.4)	0.72
Chest pain (*n*, %)	4 (7.1)	3 (6.1)	1
Syncope (*n*, %)	5 (8.9)	1 (2.0)	0.21
*Previous anticoagulant therapy*	*4 (7.1)*	*3 (6.1)*	*1*
Sudden cardiac death among family members (*n*, %)	5 (8.9)	4 (8.1)	1
TTE			
LVEDD (mm)	63.9 [40–82]	66.6 [52–80]	0.09
LVESD (mm)	53.8 [36–75]	55.8 [42–75]	0.25
LA diameter (mm)	43.5 [23–60]	42.3 [29–63]	0.49
LA volume (ml)	82.3 [30–170]	78.6 [26–163]	0.85
LVEF (%)	32.5 [15–44]	31.6 [15–44]	0.52
LVEDV (ml)	192.6 [73–360]	205.4 [90–395]	0.30
LVESV (ml)	130.5 [43.4–279]	146.2 [59–298]	0.17
High Filling Pressures (n, %)	16 (28.5%)	15 (30.6%)	0.70
Cardiac Index l/mn/m^2^	2.29 [1.15–4.95]	2.22 [1.37–4.4]	0.81

*LVEF, left ventricular ejection fraction; LVEDD, left ventricular end-diastolic diameter; LVESD, left ventricular end-systolic diameter; LA, left atrium, LVEDV, left ventricular end-diastolic volume; LVESV, left ventricular end-systolic volume*.

**Figure 3:**
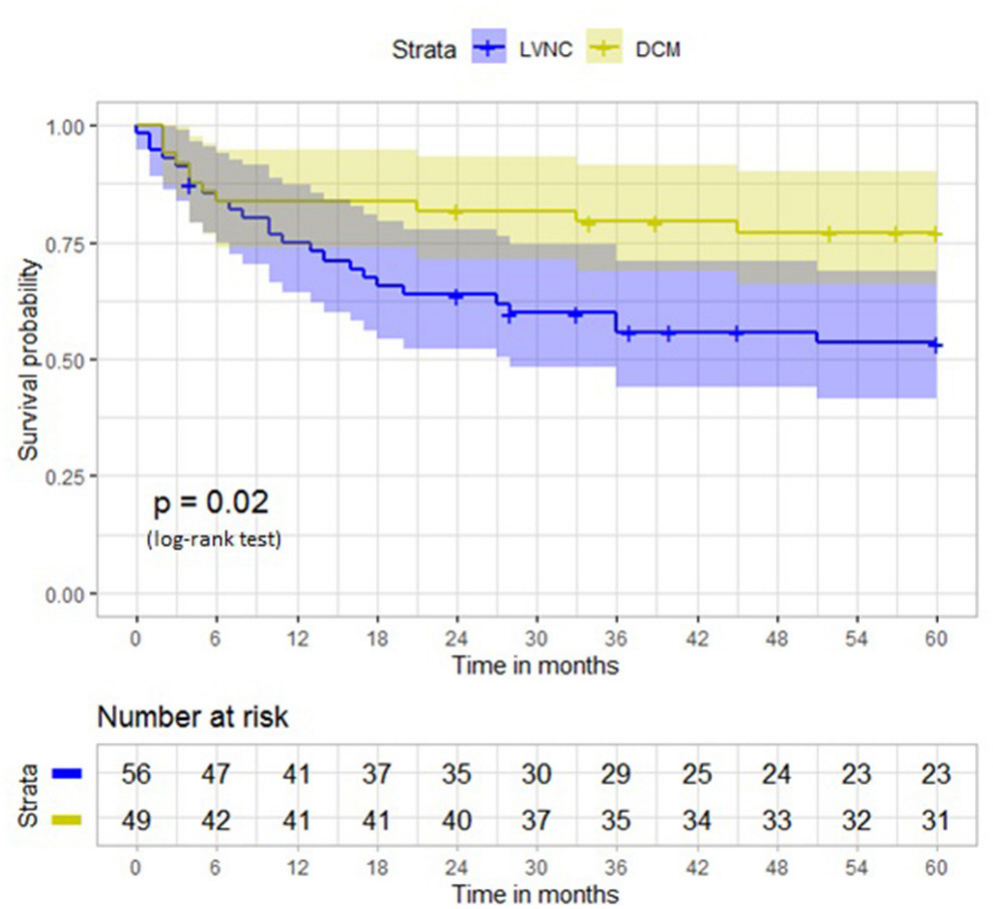
Survival free from primary outcome at 5-year follow-up in patients with left ventricular ejection fraction <45%. LVNC, left ventricular non-compaction; DCM, dilated cardiomyopathy.

At 5-year follow-up, the primary endpoint occurred in 33 (58.9%) patients with LVNC and 18 (36.7%) patients with DCM (*p* = 0.02) (Table 3). The Kaplan–Meier curve in Figure 3 illustrates the 2 groups' event-free survival.

**Table 3:** Five-year outcome.

	**LVNC with LVEF ≤ 45% *N* = 56**	**DCM with LVEF ≤ 45%** ***N* = 49**	*p*-value ^*^	**LVNC with LVEF > 45%** ***N* = 42**	*p*-value ^**^
Main endpoint, *n* (%)	33 (58.9)	18(36.7)	0.02	4 (9.5%)	0.00016
Heart failure, *n* (%)	18 (32.1)	5 (10.2)	0.03	2 (4.8)	0.04
Embolism, *n* (%)	5 (8.9)	1 (1.0)	0.21	0	0.263
Rhythmic disorders, *n* (%)	24 (42.8)	12 (24.4)	0.174	3 (7.8)	0.005
Heart Transplantation, *n* (%)	8 (14.3)	0	0.008	1 (2.6)	0.147
Cardio-vascular death, *n* (%)	4 (7.1)	4 (7.8)	1	0	0.155
Number of patients experiencing a second cardiovascular event during the 2-year follow-up, *n* (%)	28 (50.0)	8 (16.3)	0.01	4 (9.52)	0.004

* *p-value between LVNC and DCM with LVEF ≤ 45%.*

** *p-value between LVNC with LVEF ≤ 45% vs. LVNC with LVEF > 45%*.

When looking at the occurrence of the primary outcome components taken separately (Table 3), hospitalization for heart failure (18 [32.14%] vs. 5 [10.20%], p = 0.035) and heart transplantation were much more frequent in the LVNC group at a 5-year follow-up than in the DCM group. Rhythmic complications were slightly more frequent in the LVNC group, but embolic events and cardiovascular death incidences were similar. Intracardiac defibrillator implantation was performed in 18 (32.1%) patients with LVNC and 10 (20.4%) patients with DCM. The number of patients experiencing more than 1 cardiovascular event during follow-up was also higher in patients with LVNC (Table 3).

### Outcome of Patients With LVNC Depending on Their LVEF

**Figure 4:**
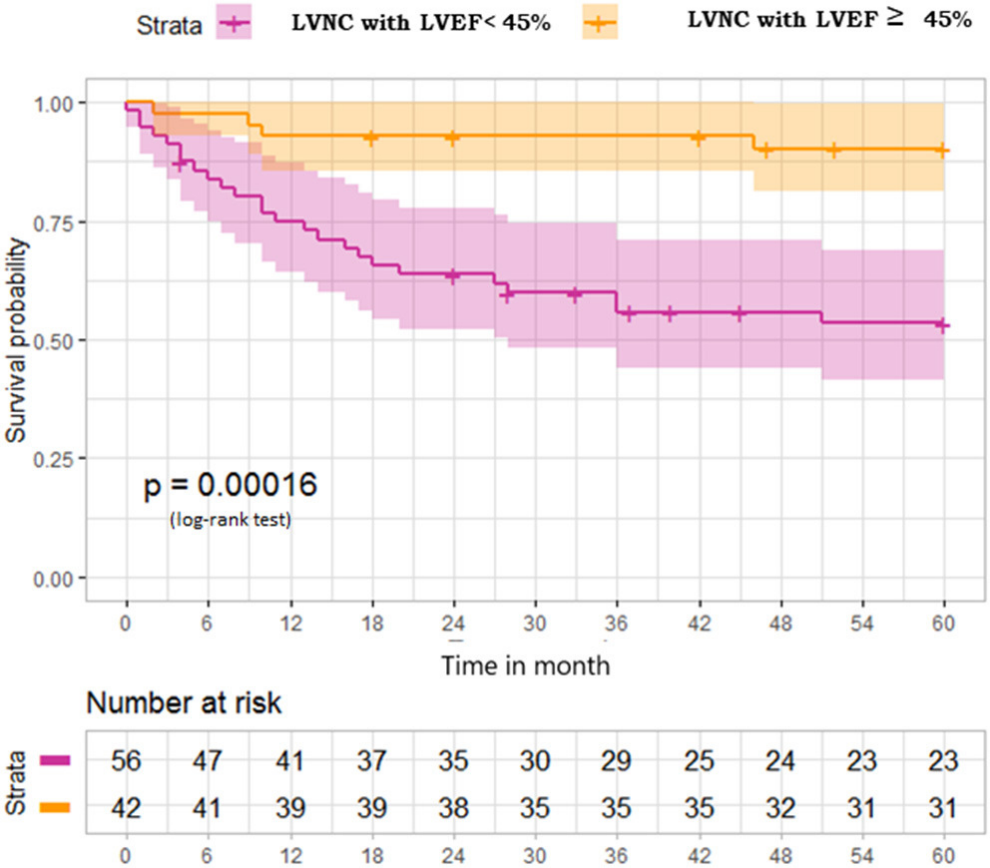
Survival free from primary outcome at 5-year follow-up in patients with LVNC depending on their left ventricular ejection fraction. LVNC, left ventricular non-compaction; LVEF, left ventricular ejection fraction.

Patients with LVNC and LVEF ≤ 45% (*n* = 56) were compared with patients with LVNC and LVEF > 45% (*n* = 42). Global longitudinal strain (GLS) values were available in 30 of 42 patients with LVNC and LVEF > 45%. GLS was relatively low in these patients (−18.1 [−22 to −13.2%], despite normal or near-normal LVEF (59.5 [47–68.4%]). Events were very rare in the latter group during the 5-year follow-up, with primary endpoints occurring in only four (9.5%) patients, including two hospitalizations for heart failure, three rhythmic complications, and no embolic events (Table 3, Figure 4).

## Discussion

### Key Findings

Patients with LVNC and left ventricular dysfunction (LVEF <45%) present with a significantly worse prognosis at a 5-year follow-up as compared with matched patients with DCM and similar LV dysfunction.

Notably, heart failure events are more frequent in patients with LVNC than in those with DCM, while embolic events are not observed.

Conversely, patients with LVNC and LVEF >45% have very few events at 5 years.

### DCM and LVNC Mortality

The prognosis of LVNC has always been debated. Initially considered as a rare cause of heart disease (<1%) affecting young patients, LVNC natural course accounted for early and frequent cardiovascular events (7, 8). Initial cohorts incriminated LVNC for up to 38% of long-term mortality. Conversely, other studies reported a higher prevalence around 3–4% (16, 17) and described LVNC as a subgroup of cardiomyopathies with no prognostic difference (10, 18, 19). The diversity of prognosis and phenotypes has been assigned to the genetic background being itself widely heterogeneous and to various definitions that hamper comparisons between cohorts. The most recent studies are again contradictory. Aung et al.'s meta-analysis over 3 years of follow-up (19) and a recent Spanish retrospective study over 5 years of follow-up compared cardiovascular mortality between LVNC and DCM and argued both for equivalent prognosis (20). However, both studies suffer from several limitations; the most important being their retrospective nature. In addition, the meta-analysis was limited by variable LVNC definitions and heterogeneous primary endpoints when the Spanish study was limited by a small population, especially a few controls with only 13 DCM. More recently, Vaidya et al. compared the long-term survival of patients with LVNC with expected survival of age- and sex-matched US population and found that patients with LVNC have a reduced survival rate. However, this study was retrospective and did not compare the prognosis of patients between LVNC and DCM (21). Sedaghat-Hamedani et al. (22) compared an age-matched population of LVNC and DCM. Among 65 LVNC and 247 DCM cases, cardiovascular events were significantly more frequent in patients with LVNC than in those with DCM (*p* = 0.002, HR = 2.481). Unfortunately, in these series, patients were matched only on age and not on LVEF.

Our study is the largest prospective cohort comparing LVNC to DCM. All centers followed the European Society of Cardiology's current recommendations, and patient care was conducted accordingly. Although the occurrence of cardiovascular death was similar in LVNC and DCM cases, the primary endpoint, including the occurrence of cardiac death, heart transplantation, or hospitalization for cardiovascular complications (hemodynamic, rhythmic, or embolic), was more frequent at 5-year follow-up in the LVNC group.

### DCM and LVNC Morbidity

Overall survival depends on the occurrence of cardiovascular events with different impacts of hemodynamic, rhythmic, and embolic factors.

#### Embolic Events

LVNC embolic risk is supposed to be related to the formation of thrombus in the trabeculations. Some authors have recommended curative anticoagulation as primary prevention even when LV dysfunction is only moderate (<45%) (23–25). Sedaghat-Hamedani et al. (22) reported up to 10% of thromboembolism, but this rate was quite different in other studies (6, 8). Like most recent publications, our results found a low incidence of thrombo-embolic events and do not support prophylactic anticoagulation (26). Such a strategy seems accurate only for patients with LVNC who have atrial fibrillation or severe left ventricular dysfunction (26). Atrial fibrillation has been reported in up to 22% of patients with LVNC (21), has been related to left atrial dilatation, which is present in 50% of cases in these series, and has been associated with a worse prognosis.

#### Hemodynamic Events

The incidence of hemodynamic events also varies but is generally very high from 25% to more than 50% at 3 years of follow-up (8, 26). In line with previous studies, it was the second most frequent event in our population, with more than 30% requiring hospitalization for heart failure during a 5-year follow-up. More importantly, heart failure complications were clearly more frequent in patients with LVNC than in those with DCM in our study. Our results are consistent with the meta-analysis of Aung et al, who found that patients with LVNC had a higher incidence of heart failure hospitalization than DCM patients (19).

#### Arrhythmic Events

Rhythmic events occurred in >40% of our population of LVNC, slightly (but not statistically significantly) more frequently than in DCM. Since the first prognostic studies that showed 18% of sudden cardiac deaths (7), rhythmic events have been increasingly recognized as LVNC's main cause of death (26). Ventricular arrhythmia has been reported in up to 41% of patients with LVNC (8, 11, 20). Almost one-third of patients with LVNC had an ICD implantation, and more than 30% of them were reported to receive appropriate defibrillation in a 3-year follow-up study (27). Arrhythmia substrate is presumed to be linked to fibrosis development in the trabeculations responsible for the ventricular reentry mechanism (28).

### Limitations

Our study suffers from some limitations. First, the diagnosis of LNVC is frequently difficult, and the differentiation between LVNC and DCM may be difficult in some patients (5, 6). However, selection criteria were very strict in this study, with validation by a core lab specialized in the echocardiographic diagnosis of LVNC (6, 12). Second, the number of events was relatively low, particularly in patients with LVEF >45%, but the combined endpoint was clearly more frequent in patients with LVNC who had LV dysfunction as compared with DCM patients. Third, cardiac MRI was not performed in all patients in our series, and the predictive value of late gadolinium enhancement and fibrosis detected by MRI could not be evaluated (29). In addition, although LVEF was calculated in all patients, GLS was not available in all patients. Interestingly, however, when measured in patients with normal or near-normal LVEF, GLS was relatively low, possibly indicating the presence of mild left ventricular longitudinal dysfunction in these patients (30). Finally, and more importantly, this study was the first prospective one to allow a head-to-head comparison of patients with LVNC and DCM, with similar age and ejection fraction.

## Conclusion

We report the largest LVNC prognostic prospective cohort with a clear trend toward poorer prognosis compared to DCM when considering patients with left ventricular dysfunction. Although cardiovascular mortality is similar between LVNC and DCM, heart failure events and, to a lesser degree, rhythmic events are more frequent in patients with LVNC than in those with DCM, while embolic events are not. Our results support the careful evaluation and prevention of hemodynamic complications as well as regular screening for rhythmic complications. Conversely, our results do not support prophylactic anticoagulation in LVNC unless a specific indication exists.

Further long-term survival studies are needed to evaluate the effects of different therapeutic strategies on the treatment of patients with LVNC.

## Data Availability Statement

The raw data supporting the conclusions of this article will be made available by the authors, without undue reservation.

## Ethics Statement

The studies involving human participants were reviewed and approved by institutional review board La Timone Hospital. Written informed consent for participation was not required for this study in accordance with the national legislation and the institutional requirements.

## Author Contributions

HG, NI, HM, KN, RG, PC, and GH were involved in the conception and design or analysis and interpretation of data. PR, ED, J-CE, OH, CS-S, PR-R, GJ, NM, CS, FA, J-FP, A-CC, NM, VD, LF, RG, PC, and GH were involved drafting of the manuscript or revising it critically for important intellectual content. HG, KN, PC, and GH were involved in the approval of the manuscript submitted. All authors contributed to the article and approved the submitted version.

## Funding

This study was supported by grants from the PHRC (projet hospitalier de recherche clinique) PHRC 2011-A-00987-34 (France).

## Conflict of Interest

The authors declare that the research was conducted in the absence of any commercial or financial relationships that could be construed as a potential conflict of interest.

## Publisher's Note

All claims expressed in this article are solely those of the authors and do not necessarily represent those of their affiliated organizations, or those of the publisher, the editors and the reviewers. Any product that may be evaluated in this article, or claim that may be made by its manufacturer, is not guaranteed or endorsed by the publisher.
